# Bowhead whale foraging dives are defined by speed and body orientation

**DOI:** 10.1371/journal.pone.0343408

**Published:** 2026-04-24

**Authors:** Manon F. den Haan, Paolo S. Segre, Eric Kilabuk, Mason Agnakak, Fred Dialla, Sarah M. E. Fortune

**Affiliations:** 1 Department of Oceanography, Dalhousie University, Halifax, Nova Scotia, Canada; 2 Department of Natural and Applied Sciences, University of Wisconsin – Green Bay, Green Bay, Wisconsin, United States of America; 3 Kilabuk Services Inc., Pangnirtung, Nunavut, Canada; Coastal Carolina University, UNITED STATES OF AMERICA

## Abstract

Quantifying the time baleen whales spend foraging is central to understanding the extent to which climate driven shifts in low-trophic prey may affect energy acquisition. To date, the most comprehensive understanding of balaenid (bowhead and right whale) feeding behavior comes from studies using inertial sensing tags (biologgers). Here, periods of ram filtration were inferred under predictable foraging conditions where whales targeted a deep, diapausing prey layer. However, foraging conditions are increasingly variable in high latitudes and zooplankton vertical distribution, abundance and biomass vary seasonally based on ontogenetic migration. Our objective was to identify the kinematic features of confirmed bowhead feeding in a sub-Arctic fjord where prey availability fluctuates with tidal cycles, by collecting high-resolution, 3D biologging data (Customized Animal Tracking Solutions Tags) and concurrent video recordings for behavioral validation. Using a mixed-model approach we found that mean bottom speed, bottom pitch and roll, change in average swim speed and time of day were the best predictors of bowhead whale feeding. Consistent with previous studies, fluke stroke frequency was significantly faster when feeding (0.191 Hz ± 0.045 SD) compared with not-feeding (0.172 Hz ± 0.037 SD). However, we observed a depth-dependent switch in fluke stroke frequency, whereby the fluking rate was higher (0.192 Hz ± 0.039 SD) at shallow depths (≤23 m) and lower (0.151 Hz ± 0.024) at deeper depths (>23 m). Observed shifts in fluke stroke frequency may be attributed to physical forces acting in the upper water column such as buoyancy, drag and tidal forces. By using the descriptive kinematic variables, we can better identify feeding behavior in kinematic data from balaenids and improve our understanding of foraging efforts of bowhead whales in a changing climate, particularly since dive shape alone may overestimate feeding activities.

## Introduction

Energy acquisition is fundamental to the survival of marine mammals as successful foraging is needed to fuel physiological processes such as metabolism, growth and reproduction [1]. However, evaluating whether animals are obtaining sufficient resources, requires accurately identifying foraging behavior. This is particularly important for polar species that are experiencing increased environmental variability and borealization of prey [2]. Balaenids (bowhead and right whales) use ram filtration to filter mostly calanoid copepods from the water [3–5]. This is a steady-state, continuous process where unidirectional flow enters the mouth and exits at the posterior opening near the eye [6,7]. Although the morphological features of balaenid mouths create hydrodynamic conditions that facilitate efficient filtration, this process creates drag and results in reduced swim speeds [5]. Brief pauses in swimming occur after several minutes of continuous fluke strokes. Such breaks in fluking have been interpreted as the whale swallowing its prey and/or cleaning its baleen [3,4]. Consequently, reductions in swim speed and increases in fluke stroke frequency and amplitude have been used to identify when ram filtration occurs [4].

Compared to feeding biomechanics of rorquals that utilize dynamic lunge-feeding strategies [3,8–11], less is known about the kinematics of ram filtration. Simon et al. (2009) and van der Hoop et al. (2019) provide the only detailed description of balaenid whale behavior and biomechanics on filter feeding to date [4,12]. They used multi-sensor Digital Acoustic Recording Tag (DTAGs) to record underwater movement and biomechanics of bowhead whales (Eastern Canada-West Greenland population) in Disko Bay (DB), Greenland and North Atlantic right whales (*Eubalaena glacialis*, hereafter NARW) in the Bay of Fundy, Canada. For bowheads, feeding was inferred based on the presence of an acoustic “rattling” sound believed to be generated by the baleen. Whereas dive shape was used to infer when NARWs were feeding. Reduced swim speed and increased fluke stroke with short pauses were observed for both species when they were thought to be feeding [4,12]. However, both studies quantified feeding mechanics for whales that were exploiting diapausing zooplankton found consistently near the sea floor [12–14]. It is unclear whether balaenids have the same kinematic signature when feeding in comparatively variable environments, as is common in Arctic fjords and whether other metrics of body position, orientation and movement may improve identification of feeding bouts.

The behavior of Eastern Canada-West Greenland (ECWG) bowhead whales (*Balaena mysticetus*) has mostly been studied using long-term satellite tags that record vertical diving behavior [14–18]. Horizontal movements with low swim speed and high turning angles along with dive shape have been used to identify putative feeding behavior [15,17,19]. Specifically, U and Square-shaped dives are thought to reflect foraging with prey ingestion occurring during the longer bottom-phase, whereas V-shaped dives indicate travel or exploration [13,14,20,21]. In our study, we sought to answer the following questions: 1) are the kinematic signatures of balaenids feeding in variable environments different from those foraging under more consistent conditions (e.g., on diapausing prey)?; and 2) do detailed kinematic measurements provide a more accurate means of identifying feeding bouts than dive profiles inferred from time-depth record (TDR) data?

To answer these questions, we visually validated kinematic data collected from bowhead whales in Cumberland Sound, NU during summer, where they have alternated between feeding on shallow and deep prey layers [22]. Customized Animal Tracking Solution (CATS) biologging tags equipped with a forward-facing camera allowed us to identify the kinematic signature of confirmed bowhead whale foraging dives. We audited the video footage using behavioral analysis software and calculated kinematic variables for each dive and dive phase (descent, bottom and ascent). Our objective was to identify the video validated kinematic features that describe bowhead whale feeding in variable environments and compare this with traditional methods of quantifying foraging efforts such as dive shape. We hypothesized that peaks in jerk could be associated with closing of the mouth, since frontal surface area decreases resulting in a decrease in drag, allowing for a brief increase of acceleration. This assumption is supported by observations of faster fluking rates during balaenid foraging during the bottom phase compared with the descent and ascent phase [4]. A Generalized Linear Mixed-Effects model was used to identify which kinematic variables best described confirmed bowhead whale feeding. We further assessed the accuracy of using dive shape to identify feeding behavior, validated through visual confirmation of feeding events.

## Methods

### Data collection

**Fig 1:**
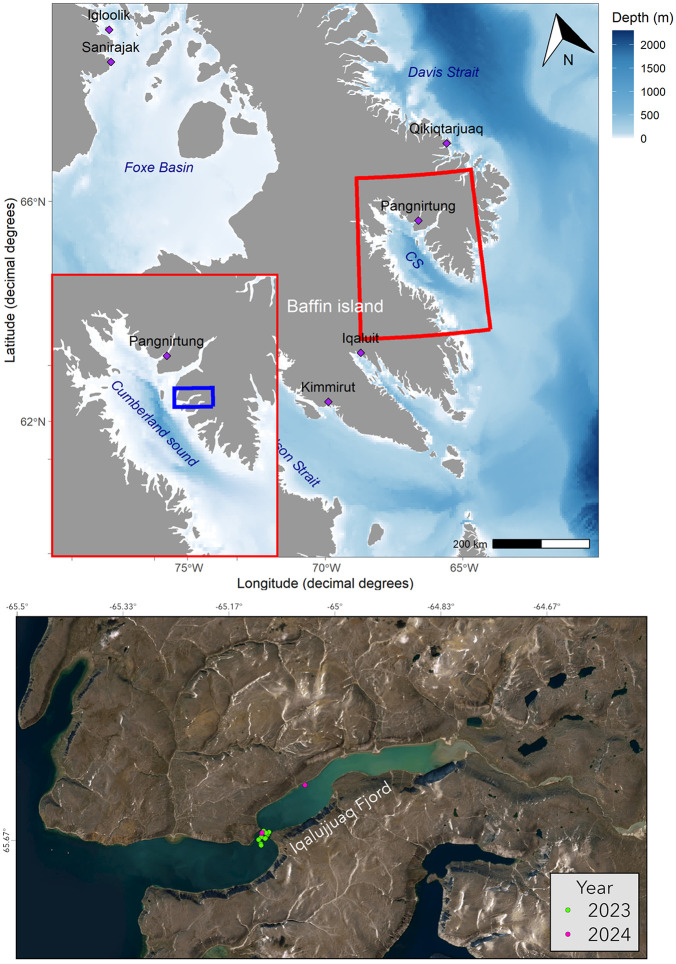
Map of all tagging locations in Iqalujjuag Fjord, Cumberland Sound (Nunavut, Canada). Green points indicate locations where bowhead whales were tagged in 2023 (n = 12) and pink points show animals tagged in 2024 (n = 3). Zoomed in figure of Iqalujjuag Fjord was rendered in ArcGIS. Figure 1 was created both in R and ArcGIS. The upper panel was created with the package “ggOceanMaps [23]. The ggOceanMapsData land polygons are from Natural Earth data, distributed under the CC Public Domain License. For ggOceanMapsData Bathymetry, NOAA Bathymetry data is used from the ETOPO 2022 15 Arc-Second Global Relief Model [24]. The lower panel map of Fig 1 was created using ArcGIS software (version 3.1.2) by Esri. ArcGIS is the intellectual property of Esri and are used herein under license. Copyright Esri. All rights reserved.

Bowhead whales were equipped with high-resolution biologgers during July and August in 2023 and 2024. Animals were tagged in Cumberland Sound, Nunavut, Canada (Fig 1). Customized Animal Tracking Solutions (CATS) tags were used to record the underwater movement with a high sampling frequency. Tags were equipped with a tri-axial accelerometer (400 Hz), magnetometer and gyroscope (50 Hz), time-depth recorder (TDR) (10 Hz) and fast-acquisition GPS. Concurrent passive acoustic and video recordings were made using an integrated hydrophone and forward-facing camera. The CATS tags were deployed using a carbon fiber pole (6 m) and attached to the whale using either suction-cups or dermal anchors. Dermal anchors were made of 7 cm long stainless-steel and were autoclaved and coated in Betadine solution prior to deployment to ensure sterilization [22,25]. Galvanic releases were used during dermal attachments to ensure tags detached after 24 hours. After detachment, tags were recovered using either: 1) built-in satellite recovery beacon that included Very High Frequency (VHF) telemetry signals; or 2) Wildlife Computers SPOT 363C satellite tags with Ultra High Frequency (UHF) telemetry signals.

### Ethics statement

The study protocol was approved by the University Committee on laboratory Animals (UCLA) of Dalhousie University (file number (22−049) Bowhead whale foraging ecology). It was also approved by the Ontario, Prairie and Arctic Animal Care Committee (OPA-ACC) of Fisheries and Oceans Canada (number: OPA-ACC-2023-23 and OPA-ACC-2024–29). No animals were euthanized to conduct this study.

### Data processing

After tag recovery, the data was processed in MATLAB version 2023a [26] using the CATS tools developed by Cade et al. [27]. This process involved several steps to ensure that the high-resolution sensors were integrated with the audio- and video-data (time-aligned). Any time offset in the video recordings was corrected to compare the start time of the deployment that was noted in the video recording to the start of the deployment in the kinematic data. After importing, all high-resolution sensor data (diary) were downsampled to 10 Hz. Next, the non-biological data (e.g., tag recording time when not attached to whale) was truncated, and the video data was synchronized with the diary data. Pressure, accelerometer and magnetometer data were calibrated using in-situ and spherical methods [27]. After calibrating, the tag orientation frame was converted to the whale orientation, and potential tag slips were identified and accounted for [28]. The last step involved the calculation of tag jiggle, a proxy for forward speed [29]. The accelerometer jiggle (at 400 Hz) was regressed against orientation-corrected depth rate (OCDR) [11,30,31]. The correlation coefficient varied between deployments (Table S1 in S1 File), likely because of variation in tag attachment time. For example, shorter deployments provided fewer accelerometer jiggle data points to regress against OCDR. In addition, the minimum detectable jiggle speed is ~ 0.9 m/s [29] and swim speeds below this threshold are less reliable than those calculated from higher speeds [29]. However, our focus was on relative comparisons of overall trends in velocity, not in absolute values.

Tri-axial accelerometer, gyroscope and magnetometer data were filtered using a zero-lag second order lowpass Butterworth filter (cut-off frequency 0.08 Hz) in R (version 4.4.1) [32]. The Butterworth filter design has been commonly used to isolate the movement of the body from the movement of the flukes in similarly sized whales [33]. This lowpass filter removed fast body movements (dynamic component), sampling error and noise in the signal [33]. Since broad scale movements across individuals was of interest, whale orientation (static component), including pitch, roll and heading, was recalculated using the filtered data [28,33]. This allowed us to remove small variances due to sampling noise and minor changes in body orientation. To measure fluke strokes, we applied a zero-lag Butterworth filter (bandpass, cut-off frequency = 0.08 > Hz < 0.2) to the gyroscope (y-axis) [34]. The gyroscope signal was then used to quantify rotational body movement along the pitch axis of the animal [34]. Since the gyroscope measured velocity, it had a phase shift compared to the pitch and therefore, zero-crossings with a positive inclination corresponded with the start and the peak of the fluke stroke [34].

### Video audit

**Fig 2:**
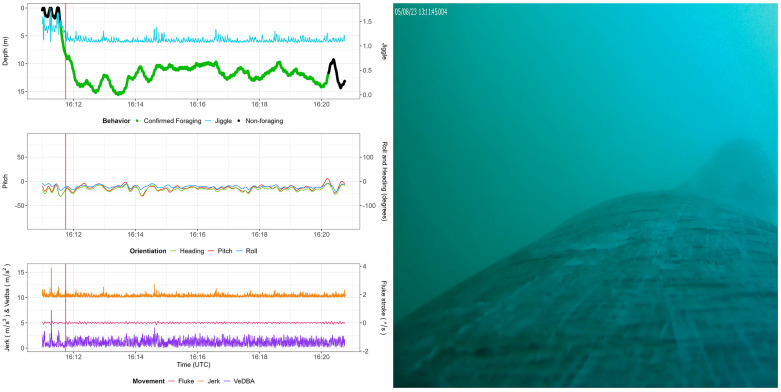
Example video of confirmed feeding behavior (August 5th, 2023) (right) and corresponding kinematic variables (left). Top left panel provides an overview of the Time-Depth Recorder (TDR), which is color coded in green for feeding behavior. The light blue shows the swim speed (m/s) of the animal. The middle panel shows the pitch (red), roll (blue) and heading (green) given in degrees, describing the body orientation of the animal. The lower panel provides an overview of movement variables: jerk (m/s^3^, orange), VeDBA (m/s^2^, purple) and fluke stroke (degrees per second based on y-axis gyroscope, pink). VeDBA has an offset of 10 units. Available at: https://drive.google.com/file/d/1XK2gTqEjdsoeNMbYKRRbzOP0zbLep68v/view?usp=sharing.

Video recordings made during tag deployments were processed using behavioral analysis software (Behavioral Observation Research Interactive Software; BORIS) [35]. In some cases, only part of the deployment was recorded. The video footage was audited for multiple behaviors and events (Table S2 in S1 File). Three behavioral events were recorded: “Foraging”, “Potential foraging” and “Travel”. For forward-facing recordings where the tagged whale’s mouth was visible, we labelled instances to be “Confirmed Foraging” (hereafter known as feeding) when the whale's mouth was open. With “Potential Foraging”, the mouth was not clearly visible, but based on behavioral clues, feeding was suspected. For confirmed ‘Travel”-events (hereafter known as non-feeding) we did not have a high degree of confidence that the mouth was agape, nor did we see zooplankton or feeding conspecifics. Combined, the time stamps of these events were used to determine the kinematic features of foraging (Fig 2).

### Calculation of movement variables

All tag deployments were separated into individual dives based on threshold depth and duration values. A dive included the following features: excursions deeper than 5 meters that lasted a minimum of 10 seconds, with a post-dive surface interval greater than 10 seconds. For every dive, the start and end of the bottom phase was manually determined by examining the dive profiles using a shiny application in the “bustR” package [36]. The descent phase began once the whale exceeded 3m and ended when the whale reached the start of the bottom phase. The ascent phase began at the end of the bottom phase and ended when the whale reached 3m. Once the dive phases had been manually identified, kinematic variables were calculated for each phase (descent, bottom, ascent) that were thought to be behaviorally important (Table S3 in S1 File). The start and end of the dive were determined in 24-hour UTC time and the duration of the dive was calculated in minutes.

We used a hierarchical process to match tagged whale behaviors from video audits with the corresponding dive data. If only one behavior was observed, that behavior was assigned either to the entire dive (descent, bottom and ascent) or the corresponding dive phase. If more than one behavioral state (e.g., “Feeding” and “Potential feeding”) was assigned to a given dive, priority was always given to “Feeding”, independent of the time spent in that behavioral state. If two or more behaviors were present and “Feeding” was not included, we selected the behavioral state with the longest duration. Only in cases where there was no video for the entire duration of the dive or dive phase, a “no-video” label would be assigned.

After assigning behavioral states to our dive data, we calculated various kinematic variables that occurred over the corresponding time-period. We calculated the mean ascent and descent rates (m/s). Fluke stroke frequency was estimated as the inverted mean of time between fluke stroke peaks. The fluke rate represents the total number of fluke strokes within a rolling 30-second period. Jerk, speed and the dynamic body acceleration are all measures of the jiggle of the accelerometer, however they capture different attributes of movement. Jerk is the combined differential of all three-accelerometer axes (i.e., x, y, z) and is useful for identifying small and rapid movements [37]. Jerk is calculated as the vectorized sum of the difference between consecutive acceleration values in each axis. To convert from acceleration in g-forces back into SI units of m/s^2^, we multiplied by the gravitational force of 9.81 m/s^2^ (Equation 1).


$$Jerk=\frac{\sqrt{\overset{\text{2}}{\underset{x}{\Delta a}}+\overset{\text{2}}{\underset{y}{\Delta a}}+\overset{\text{2}}{\underset{z}{\Delta a}}}}{sampling\;frequency}*\text{9}.\text{81}$$ (1)

The peak in jerk was calculated as the max value for jerk during a given dive phase, subtracted by the median jerk during the same phase, allowing us to account for intra-individual differences in jerk.

Fluke rate change was calculated as the difference between the mean fluke rate 20 seconds before and after the peak in jerk. The Vectorial Dynamic Body Acceleration (VeDBA) which is used as a proxy for energy expenditure (Equation 2), was calculated with the dynamic component of the accelerometer (a_dyn_) in all three axes as described above, by the zero-lag second order Butterworth filter on the raw accelerometer data [38–40].


$$VeDBA=\sqrt{\overset{\text{2}}{\underset{x_{dyn}}{a}}+\overset{\text{2}}{\underset{y_{dyn}}{a}}+\overset{\text{2}}{\underset{y_{dyn}}{a}}}*\text{9}.\text{81}$$ (2)

The assumption is that VeDBA (m/s^2^) would increase during foraging bouts since whales must overcome the drag that is increased when their mouth is open. For body orientation, both the mean roll and pitch, roll at time of peak jerk, and the heading variance was calculated. The mean swim speed was based on the jiggle of the tag and the change in speed was calculated 20 seconds before and after the peak in jerk.

To calculate the time of day (TOD), we used the start time of each dive and converted it into decimal hours. Next, we converted from a linear to a cyclical state of time using the following:


$$Time\;of\;Day=cos\left(\frac{\text{2}\pi \cdot time}{\text{24}}\right)+sin\left(\frac{\text{2}\pi \cdot time}{\text{24}}\right)$$ (3)

Due to the high latitude of the study area and time of year, the sky remained partially illuminated by the sun (i.e., twilight). During deployment days, the average time of sunset was 01:32:30 UTC and sunrise was at 07:28:30 UTC. While the sun did set, complete darkness did not occur during our study period. We know from other whale species that day/night plays a role in their foraging behavior [41,42].

Since dive shape is often used to infer balaenid whale foraging [14,18,21], we categorized each dive as V, U or Square based on the proportion of the total dive duration that was spent in the bottom phase. V-shaped dives included those with a bottom time of 20% or less of the entire dive duration. Whereas probable foraging U and Square-shaped dives included those where bottom time was > 20% and ≤50% (U-shaped) and ≥50% of the whole dive duration (Square-shaped) [18,43].

### Statistical analysis

To determine which variables best predicted the kinematic signature of bowhead whale foraging, we used a mixed modelling approach in R with the “lme4” package [44]. Using the results from our video audits, we could assign a behavior to each dive. However, due to noise in the kinematic variables classified as “Potential foraging”, we excluded those dives from our analysis and only used dives associated with “feeding” or “non-feeding”. We compared the kinematic variables using a Generalized Linear Mixed-Effects Model (ab. GLMM, “GLMER”-function in lme4 package) with a binomial family (link = “logit”), where dive type (Feeding (1) and non-feeding (0)) as the response variable was fitted. For fixed effects, single and multiple combinations of variables were tried, with a focus on kinematic variables that coincided with the bottom phase. We included deployment ID as a random effect to account for differences between individual whales and tag placement on the body as well as within and between years. Sex was not considered, since not all individuals were of known sex. Model validation was evaluated by visualizing the residuals against the fitted values to confirm model assumptions (e.g., normality, dispersion, outliers) were met [45]. Model selection involved a-priori selecting behaviorally and biomechanically important variables and stepwise forward selection (increasing the model), while meeting model assumptions. Akaike information criterion (AIC) and logarithm likelihood values were used for model selection whereby the model with the lowest AIC and highest log likelihood was selected as the final model.

Since tidal cycle appears to influence both zooplankton presence and density and bowhead whale dive depth in Iqalujjuag Fjord [46] we evaluated whether fluke stroke frequency changed with dive depth. This comparison was made using all recorded fluke strokes from all deployments (n=15), including those without video data. Fluke strokes (n=30,439) were pooled across all individuals, dive phases (descent, bottom and ascent) and behaviors (feeding and not feeding). Fluke stroke frequencies were further grouped into 1-m depth bins. Next, a linear mixed-model [47] with Time of Day as a random variable and depth strata (shallow or deep) as a fixed variable was used. The fluke stroke frequency was standardized to account for the variability between individuals and any outliers were removed.

## Results

### Tag deployments

Overall, 23 bowheads were equipped with CATS tags during summer 2023 and 2024. However, 8 deployments were excluded from our analysis due to short deployment times (e.g., < 5 min). Consequently, our analysis included 15 CATs deployments with an average recording time of 3.84 hours (± 6.43 SD) (Table 1). Combined our tags provided 57.54 hours of high-resolution kinematic and dive data (S4 Table in S1 File). Out of the 15 deployments, 11 were attached with suction cups and 4 with dermal anchors. One of our tags (P49) had a malfunctioning camera, resulting in no video recordings that could be used for behavioral audits to validate feeding events. Consequently, we excluded CATS data without corresponding video recordings (n = 5 deployments) from our GLMM model (Table S4 in S1 File). After removing kinematic data that lacked corresponding video recordings, we had ten deployments with a cumulative total of 9.43 hours of video footage collocated with diary data that was used in the GLMM.

**Table 1:** Dive summary statistics (mean and standard deviation) of deployments where Pitch, Roll and Heading (PRH) files could be created.

ID	# of dives	Maximum dive depth (meters)	Dive duration (minutes)	Descent duration (minutes)	Bottom duration (minutes)	Ascent duration (minutes)
av230803-180a	4	15.13 ± 9.43	4.45 ± 3.63	0.20 ± 0.09	3.98 ± 3.48	0.27 ± 0.23
av230803-P48_a	1	13.86	1.38	0.31	0.70	0.37
av230803-P48_b	16	18.06 ± 10.55	4.85 ± 3.79	0.68 ± 0.72	3.59 ± 3.77	0.58 ± 0.41
av230803-P49	15	23.42 ± 13.26	6.22 ± 3.31	0.47 ± 0.23	5.30 ± 3.34	0.44 ± 0.31
av230805-P46_a	2	10.97 ± 3.34	1.54 ± 0.05	0.26 ± 0.03	1.06 ± 0.00	0.21 ± 0.07
av230805-P46_b	23	21.32 ± 15.74	7.78 ± 5.32	0.58 ± 0.41	6.68 ± 5.05	0.52 ± 0.44
av230805-P49_a	12	22.90 ± 15.11	2.38 ± 1.68	0.40 ± 0.35	1.52 ± 1.47	0.49 ± 0.33
av230805-P49_b	5	30.33 ± 26.88	2.75 ± 1.19	0.66 ± 0.32	1.37 ± 1.04	0.72 ± 0.56
av230806-P48	114	55.43 ± 42.94	10.27 ± 5.09	0.88 ± 0.62	8.59 ± 4.27	0.82 ± 0.65
av230806-P49_a	11	16.77 ± 3.79	6.74 ± 3.28	0.34 ± 0.23	6.05 ± 3.20	0.35 ± 0.18
av230806-P49_b	8	40.29 ± 25.64	7.23 ± 2.40	1.09 ± 1.32	5.69 ± 1.67	0.66 ± 0.53
av230810-P46	17	24.76 ± 19.54	4.97 ± 3.60	0.64 ± 0.67	3.92 ± 3.48	0.46 ± 0.40
av240809−68	3	7.58 ± 2.53	0.79 ± 0.40	0.16 ± 0.17	0.46 ± 0.47	0.16 ± 0.18
av240809-P47	62	42.85 ± 39.23	10.61 ± 7.54	1.22 ± 1.23	8.05 ± 6.69	1.08 ± 1.21
av240812-P48	3	26.59 ± 25.72	5.15 ± 6.67	0.32 ± 0.23	4.26 ± 5.96	0.57 ± 0.53
Average	19.73 ± 30.11	38.51 ± 36.65	7.96 ± 5.84`	0.79 ± 0.78	6.49 ± 5.12	0.72 ± 0.74

### Dive analysis

In total, we analyzed 296 dives from 15 deployments. While there were individual differences, on average our tag deployments recorded 20 (± 30 SD) dives per individual. The majority of the dives occurred in the photic zone (<60 meter), with durations of 8 mins (± 5.84 SD). Descent and ascent duration of all individuals were very similar and on average both accounted for 20% of the dive duration.

Of the 296 dives, 74 were observed with video footage and 222 had no associated video. In total we observed 258 Square-shaped dives (87.2%; with video n = 55, without video n = 203), 31 U-shaped (10.5%; with video n = 12, without video n = 19) and 7 V-shaped (2.4% with video n = 7, with video n = 0). Most dives occurred in the top 30 meters of the water column (Table 2).

**Table 2:** Mean value and standard deviation for the max depth of each bowhead whale dive per dive shape (n = 296) from CATS tag deployments in Iqalujjuaq Fjord during summer 2023 and 2025, grouped together for dives with (Video) and without (No video) corresponding video data.

	Square (n = 258)	U (n = 31)	V (n = 7)
Video	25.8 m ± 23.35 m	16.9 m ± 14.30 m	21.4 m ± 13.26 m
No video	51.7 m ± 42.61 m	24.81 m ± 21.40 m	–

### Dive metrics and behavior

**Fig 3:**
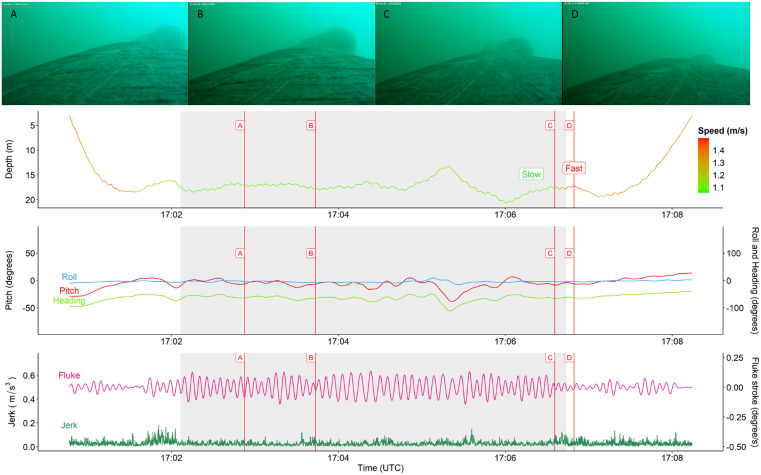
Example of a confirmed foraging dive for av230805−46 (Deployment ID) based on a video audit from August 5th, 2023. Still images (A-D) were screenshots from the CATS video recorder and correspond with vertical lines (A-D). Grey shaded areas in graphs reflect periods of confirmed feeding, based on the video audit. The top panel provides an overview of the time-depth recorder (TDR), which is colored for the speed of the animal, where green is lower and red are higher values for speed (m/s). The middle panel reflects an overview of the body orientation of the individual, where (body) Roll (blue), Pitch (red) and Heading (green) are given in degrees. The lower panel gives an overview of movement variables including jerk (m/s^3^) (dark green) and fluke stroke (degrees per second based on y-axis gyroscope) (pink).

**Fig 4:**
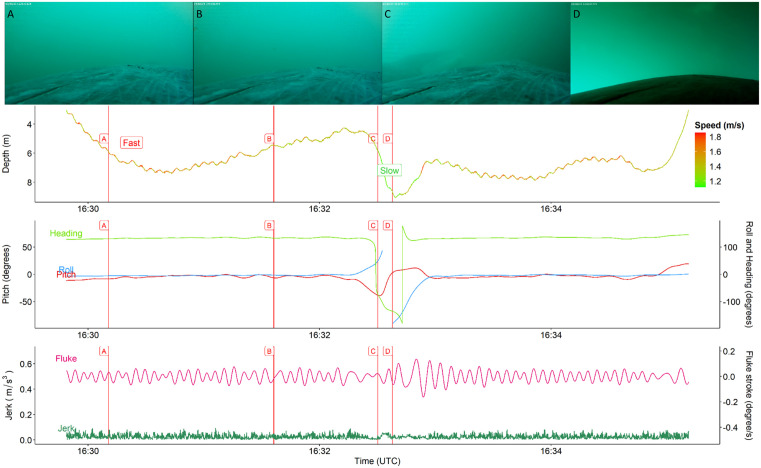
Example of confirmed non-foraging dive for av20230803-P48 on August 3rd, 2023. Still images (A-D) were screenshots from the CATS video recorder and correspond with vertical lines (A-D). The top panel provides an overview of the time-depth recorder (TDR), which is colored for the speed of the animal, where green is lower and red are higher values for speed (m/s). The middle panel reflects an overview of the body orientation of the individual, where (body) Roll (blue), Pitch (red) and Heading (green) are given in degrees. The lower panel gives an overview of movement variables including jerk (m/s^3^) (dark green) and fluke stroke (degrees per second based on y-axis gyroscope) (pink).

We found that the dive shape was not a good indicator for behavior. In both feeding and non-feeding dives, most dives were Squared-shaped (80% for feeding and 62.5% for non-feeding dives) (Figs 3 and 4). Followed by U-shaped (14% for feeding and 25% for non-feeding) and V-shaped dives (6% for feeding and 12.5% for non-feeding). This finding contradicts the assumption that Square and U-shaped dives reflect feeding [15,17,19]. Additionally, while we do see that the confirmed-feeding dives have longer durations and as whales spend more time in the bottom phase, the maximum dive depth between the two types of dives were both above 30 meters.

### Fluke stroke and swim speed

In total, 2317 fluke strokes were registered during video validated feeding and non-feeding dives, with an overall mean fluke frequency of 0.186 Hz (± 0.046 SD). The mean fluke stroke frequency when feeding behavior was observed during the descent and ascent was 0.170 and 0.168 Hz, respectively (descent: ± 0.049 SD, n = 107 and ascent: ± 0.048 SD, n = 176; Table 3). The mean fluke stroke frequency for feeding during the bottom phase was 0.191 Hz (±0.045 SD, n = 1696), which was not significantly higher than during the ascent and descent (Welch ANOVA on standardized fluke stroke frequency, p-value= 0.256). For non-feeding dives, mean fluke stroke frequency during the bottom phase was 0.172 Hz (± 0.037 SD, n = 219), which is significantly lower than during feeding (linear regression on standardized fluke stroke frequency, p-value= 0.030). The frequency of non-feeding descent fluke strokes was 0.188 Hz (±0.071 SD, n = 52), and the ascent frequency was slightly lower at 0.171 Hz (±0.052 SD, n = 39). There was no difference between any of the phases for non-feeding behavior.

**Table 3:** Summary of dive metrics for feeding and non-feeding dives as audited by behavioral software analysis (BORIS).

	Feeding	Non-feeding
Total dives (n)	50	24
Max depth (meter)	24.73 ± 23.41	21.52 ± 17.17
Dive duration (minutes)	6.54 ± 4.56	4.72 ± 4.59
Descent duration (minutes)	0.77 ± 0.66	0.83 ± 0.83
Bottom duration (minutes)	5.09 ± 4.24	2.94 ± 3.31
Ascent duration (minutes)	0.75 ± 1.02	0.94 ± 1.31
Descent Fluke stroke frequency (Hz)	0.170 ± 0.049	0.188 ± 0.071
Bottom Fluke stroke frequency (Hz)	0.191 ± 0.045	0.172 ± 0.037
Ascent Fluke stroke frequency (Hz)	0.168 ± 0.048	0.171 ± 0.052
Square (n)	40	15
U (n)	7	6
V (n)	3	3

We found that fluke stroke frequency was impacted by dive depth, independent of behavior and dive phases. Fluke stroke frequency recorded shallower than 23 meters (linear mixed-model, p-value <0.001) was significantly different from fluke strokes recorded at deeper depths (>23 meters). The median fluke stroke frequency was 0.179Hz (± 0.0004 SE) for dives shallower than 23 m and 0.172Hz (±0.0002 SE) for those occuring below 23 m.

**Fig 5:**
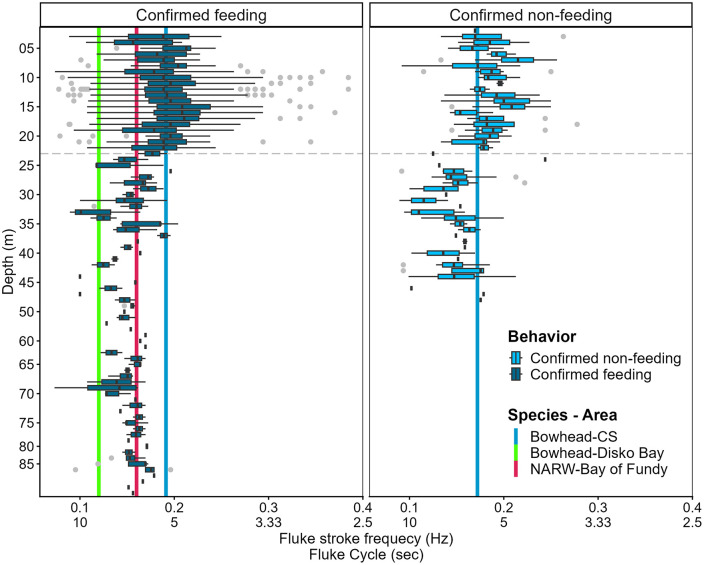
Summary of fluke stroke frequency per depth for bowhead whales (n = 10) for both feeding and non-feeding behavior based on video audits in BORIS. Vertical lines reflect the mean values of bowhead whales from our 2023-2024 study in Cumberland Sound (CS), blue. Other vertical lines are published frequency values for feeding behavior during the bottom phase of U-shaped dives (for bowhead whales in Disko Bay, Greenland have a mean fluke stroke frequency of 0.12 Hz and speed of 0.8 m/s ([4], green) and North Atlantic right whales (NARW) in the Bay of Fundy, Canada have a mean fluke stroke frequency of 0.16 Hz and speed of 1.1 m/s ([12], pink). The dashed horizontal line represents the 23-meter threshold where there appears to be a switch in fluke stroke frequency.

When accounting for dive phase, we found that fluke stroke frequency was lower (0.151 Hz ± 0.024 SD) when the bottom phase was deeper than 23 m, compared to shallower dives (0.192 Hz± 0.039 SD) (Fig 5). Furthermore, when incorporating behavior (camera validated), we found that fluke strokes decreased from 0.195 Hz (± 0.044 SD) in shallow water to 0.152 Hz (± 0.023 SD) in deep water on average during feeding bouts.

### Dive kinematics

We used multiple models to evaluate which kinematic variables ‘best’ predicted bowhead whale feeding (models NULL to 4;Table 4). Based on the AIC and log likelihood (AIC: 59.9 and log likelihood: −22.0), model 4 ‘best’ predicted feeding and included the following fixed effects: mean bottom speed, peak in jerk during the bottom phase with the change in speed, mean pitch and roll during the bottom phase and time of day (Table 5). While we recognize that the peak jerk is not yet significant, we anticipate that with a larger sample size it likely would be significant.

**Table 4:** Overview of Generalized Linear Mixed Effect Models used to ascertain the kinematic signature of bowhead whale feeding.

Model	Fixed effects	AIC	Log likelihood	ΔAIC
NULL	-	88.1	-42.1	-
1	Bottom speed	81.9	-34.5	6.2
2	Bottom fluke rate	86.0	-40	2.1
3	Bottom speed + bottom peak jerk + Bottom pitch + bottom roll + linear Time of day	66.3	-26.2	21.8
4	Bottom speed + bottom peak jerk + bottom speed change + bottom roll + bottom pitch + circular time of day	59.9	-22.0	28.2

In all models, the fixed effects were significant and did not violate the assumptions of a GLMM. Random effects (deployment ID) were consistent across the models. The changes in AIC (ΔAIC) were all relative to the null model.

**Table 5:** Results from the Generalized Linear Mixed Effects Model where dive behavior (feeding or non-feeding) was the response variable.

Fixed effect	Estimate	Standard error	Z-value	P-value
**Intercept**	8.7376	3.5651	2.451	0.01425 *
**Bottom mean speed**	−10.4117	3.5200	−2.958	0.00310 **
**Bottom peak jerk**	6.9317	3.6294	1.910	0.05615
**Bottom mean pitch**	−0.4188	0.1540	−2.719	0.00654 **
**Bottom mean roll**	0.3952	0.1590	2.485	0.01294 *
**Bottom mean speed change**	5.0429	2.5420	1.984	0.04728 *
**Time of day**	−5.5456	2.3418	−2.368	0.01788 *

Significant variables (p-value < 0.005) are indicated with an *.

**Fig 6:**
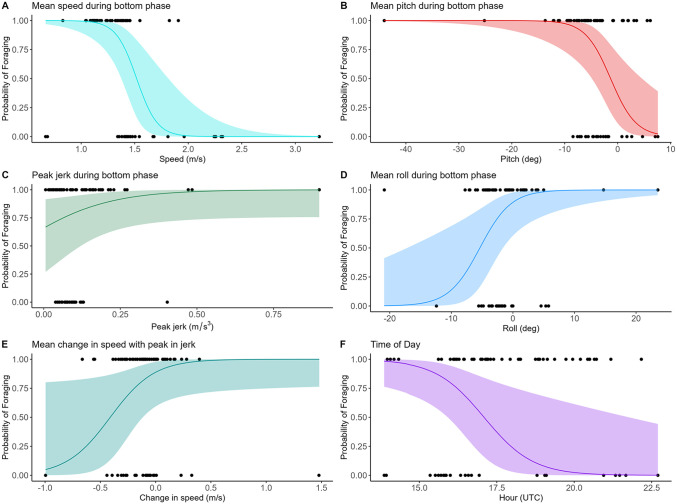
Results of Generalized Linear Mixed Effects Model 4. Where 1 includes dives with confirmed foraging and 0 reflects non-feeding dives on y-axis. (A) Mean speed during bottom phase. (B) Mean value for pitch during the bottom phase. (C) Peak value of jerk during bottom Phase. (D) Mean value for roll during the bottom phase. (E) is the mean change in speed that corresponds with the peak value for jerk and (F) is the time of day the dives started. Black points are recorded values for each variable during the bottom phase of feeding and non-feeding dives.

We found that swim speed was slower during the bottom phase of feeding dives compared to non-feeding dives (e.g., travel). Average bottom phase swim speed was 1.24 m/s (± 0.191m/s) while feeding compared to 1.64 m/s (± 0.542 m/s) during non-feeding (Fig 6A). While the peak values of jerk were not significant, we observed a slight increase during foraging dives (Fig 6C). The corresponding change in the average speed increased with peaks in foraging dives (Fig 6E). In the case of non-feeding dives, the value of peak jerks was lower, which was concurrent with a decrease in average swim speed (Fig 6C and E). On average, body pitch was slightly negative (Fig 6B) during the bottom phase of foraging dives. While individuals were pitched downward by 6.2 degrees (± 7.68 SD) on average, they also exhibited a small (10 degree) off-axis body roll that ranged from left to right.

Interestingly, time of day appeared to be driving some of the bowhead whale foraging behavior (Fig 6F), where the probability of a foraging dive decreased throughout the day. During our study, sunset occurred on average at 01:32:30 UTC and sunrise occurred at 07:28:30 UTC. Consequently, all deployments that were included in the GLMM occurred during daylight, not considering cloud cover. This implies that some circadian rhythm is driving foraging behavior independent of sunlight.

## Discussion

Our present knowledge of balaenid feeding comes from bowhead and right whale tagging studies that use dive shape to estimate foraging efforts [4,13,14,22]. Using validated kinematic data (video validated feeding events), we find that dive shape is not the best predictor of feeding behavior in highly dynamic areas. Instead, we found that a combination of kinematic variables: decreased and changes in swim speed, body pitch and roll, peaks in jerk and time of day better separates feeding from non-feeding behavior. Decreases in velocity during the bottom phase of foraging dives has already been reported in previous kinematic studies of balaenids [4,12,21]. In addition, we observed increases in speed that coincided with peaks in jerk, which may result from the closing of the mouth during feeding bouts. Furthermore, while there is individual variation, the body orientation of the whale appears to be an important feature of feeding. During foraging dives, tagged whales were slightly pitched downward towards the sea floor, yet while not feeding, whales maintained a more horizontal orientation.

### Fluke stroke frequency

Kinematically, changes in fluke stroke have been used to identify periods of ram filtration. Previous studies [4,12] reported increased fluke stroke frequency during the bottom phase of U-shaped dives (0.16 Hz for NARW and 0.12 Hz for bowhead whales) compared to the descent phase. These reported values are notably lower than the recorded fluke stroke frequency of 0.191 Hz during the bottom phase of feeding bouts for bowheads in our study (Fig 5). A possible explanation for differences in fluke stroke frequency between studies is the size of the tagged individuals. While Simon *et al*. did not report details (body length, age, sex) regarding their tagged individuals, bowheads in Disko Bay are known to be predominantly large, adult females (Adult: 80% and F:M 85%:15%) [4,14,48]. Christiansen *et al*. reported body lengths of 13.00–16.38 meters for adult bowhead whales in Disko Bay [49]. The NARWs from van der Hoop et al. had longer body lengths (average 12.04 meters) than our tagged individuals (average of 10.37 meters), which we know are juveniles and subadults (Table S4 in S1 File) [12]. Considering the hiatus in girth growth for juvenile bowheads, it’s possible the NARWs were overall bigger in size [50,51]. Due to the increased relative size of the bowheads in Disko Bay and NARW, we’d expect tagged whales from these studies to have slower fluke stroke frequencies, since fluke stroke frequency is correlated with body mass of individuals, where larger animals have lower dominant fluke stroke frequency [52].

Fluke stroke frequency is an important indicator of animal behavior and the physical forces that act on individuals. Increased fluke stroke frequency or continuous fluking has been correlated with feeding behavior, while fluke-and-glide behavior has been observed during descent and ascent, for negatively or positively buoyant species respectively [4,12,53]. We found that fluke stroke frequency varied with depth, whereby whales switched from a higher fluke gait (0.192 Hz ± 0.039 SD) above 23 meters to a slower fluke stroke frequency (0.151 Hz ± 0.024 SD) below this depth. Focusing on fluke stroke frequency during feeding dives, we observed that the mean fluke stroke frequency during inferred feeding dives reported by van der Hoop *et al.* (0.16 Hz ± 0.20 SD) falls within the variability of our observed fluke stroke frequency (mean 0.152 Hz ± 0.0230; Fig 5). The observed foraging dives of NARW’s in the Bay of Fundy were all deeper than 50 meters and there was no foraging activity in shallow waters, unlike in our study [12]. This indicates that both bowhead whales in CS and NARW’s in Bay of Fundy experience similar magnitudes at depth, but in shallow water the fluke stroke frequency independent from behavior is likely being influenced by drag forces (e.g., waves) which are greater at the surface [30]. Since bowhead and NARWs are both positively buoyant, they perform their most powerful fluke strokes during the beginning of a dive to overpower the buoyancy force [53]. This could play an additional role in the increased fluke stroke frequency observed in shallow water in our study since they must overcome both higher drag and buoyancy forces [54].

The impact of wave drag decreases with depth and loses its effect at depths more than three times the body diameter of a whale [55]. Since our whales are mostly juvenile and sub-adult males, we expect that their girth (i.e., diameter) would be between 4.0 and 4.8 meters [56]. Assuming a body girth of 4.8 m (2.4 m radius), the wave drag would cease to exist when bowheads dive below 14.4 m, which is shallower than the average depth at which fluke stroke frequency changes for our tagged whales. However, the wave drag force was calculated only considering waves created due to wind stress. IQ is a small fjord that has a semidiurnal tidal cycle with two high and two low tides per lunar day. During the days of tag deployment, the water fluctuated by 4.85 m on average (low tide: 1.5 meter ± 0.8, high tide: 6.4 meter ± 0.73, data from https://www.tides.gc.ca/en/stations/4029). Due to the relatively small opening (mouth of fjord), shallow bathymetry and multiple bottlenecks in the physical morphology of the fjord, the tidal forces are high in the top of the water column. This could explain why bowheads had a higher fluke stroke frequency in the top water column. Tidal forces may also help explain the pauses in fluke stroke we observed in deeper dives (S2 Fig in S1 File), that were absent in shallower dives.

### Dive kinematics

Previously identified pauses in balaenid fluking stroke found during the bottom phase of foraging dives were thought to reflect periods of baleen plate cleaning and prey ingestion [4,57]. However, our statistical models (GLMM) were not suited to detect such pauses and other types of signal processing, like autoregressive models might be better suited [12]. Consistent with previous studies, we observed a clear decrease in swim speed during the bottom phase of foraging dives (1.15 m/s ± 0.17 SD; 0.83 m/s to 1.83 m/s). Conversely, swim speed was consistently higher during the bottom phase of non-foraging dives (1.46 m/s ± 0.43 SD). Although statistically insignificant, the peak in jerk was higher for foraging (0.141 m/s^2^ ± 0.148) than non-foraging (0.094 m/s^2^ ± 0.070) dives on average. Increases in the accelerometer differential may indicate periods when tagged whales closed their mouth during feeding bouts. When the mouth is closed or the aperture decreases, the surface area of the whale decreases causing a small increase in the acceleration of the animal, which results in a coincident increase in mean swim speed shortly after the mouth is closed. An explanation for instances when we see less consistent and repeatable patterns in peak jerk includes physical interactions between conspecifics that bump the CATs tag, causing sudden spikes in all inertial- and depth-sensors. Although we did observe multiple conspecifics feeding simultaneously with tagged individuals, the pattern in the jerk signal was too repetitive to have been caused by species interactions alone (Figs 3 and S2 in S1 File).

Body pitch and roll are important components of the kinematic signatures for lunge-feeding in fin and blue whales [11,58]. However, for bowhead whales that employ continuous ram-filtration, we didn’t expect these variables to signify feeding [3]. Fin whales approach prey patches with a slight downward angle (~30 degrees), perhaps to lower the pressure on the underside of the head through the Bernoulli effect [11,59]. By lowering the pressure on the underside of the jaw, it is easier to open the mouth, which could also play a role for bowhead whales. We found that bowheads had a body value that ranged from −20–20 degrees across all behaviors (feeding and non-feeding). Minor shifts in body roll of −5.4 degrees were associated with a higher probability of feeding (0.5), which could be an artifact of a small sample size. Other measurements such as variance, max and min of the body roll would be more biologically significant, since it would capture larger shifts in body orientation.

Unexpectedly, variance in heading was not a significant variable in our models, both as a single explanatory variable and in combination with others. This finding contrasts with our expectation that when bowhead whales are feeding, they would have more tortuous movements to remain within profitable spatial limits of a prey patch [60]. However, the variance in heading during the bottom phase of feeding and non-feeding dives was not statistically different.

It is commonly accepted that foraging animals incur higher energetic costs [4,61]. Due to the increased drag and continuous fluking while feeding, we expected that VeDBA would be increased with respect to non-feeding dives, since VeDBA is a proxy for energy expenditure [38,39,62]. However, our models failed to detect a difference which might suggest that our method did not fully capture the nuances in the dynamic body acceleration. A recent study found that for marine mammals with body lengths greater than 3 meters, dynamic body acceleration can be influenced by body rotations (e.g., roll and fluke stroke) [63]. Martín López *et al.* (2022) suggest that the energy expenditure can only be calculated by the dynamic component if the orientation component and axis rotations are completely removed. Since the goal of our study was to examine broad kinematic signatures and was not focused on quantifying energy expenditure, the filtration method used to separate the static and dynamic components of the accelerometer may have been too coarse to obtain the best estimate for VeDBA.

A commonly described feature of balaenid feeding are deep stereotypical dives near the sea floor [4,12,14,18]. Such is the case in Disko Bay (Greenland) where tagged bowheads consistently dove to 100 m on average to feed on diapausing (i.e., overwintering at depth after accumulating sufficient lipids) life-stages of *Calanus finmarchicus* (copepods) [14]. Similarly, North Atlantic right whales tagged in the Bay of Fundy (Canada) consistently dove near the bottom mixed-layer (between 80 and 175 m) where high concentrations of late-stage (CV) *C. finmarchicus* were similarly undergoing diapause [13]. More recently, bowheads were thought to conduct putative foraging dives at shallow (22.5m ± 4.51 SD) and deep depths (260.42 m ± 35.83 SD) in Cumberland Sound (Canada) during summer [22]. Based on dive-shape and two-dimensional tag data, bowheads appeared to allocate most of their time to deep feeding and only occasionally exploited near surface prey aggregations (17). Our results suggest that balaenid feeding can be more variable than previously thought [4,14].

In both Disko Bay (DB) and Kingnait Fjord (KF, Cumberland Sound), bowheads performed repeated, deep foraging dives (inferred from dive shape), to 100 m on average to feed on high densities of diapausing copepods near the seafloor [4,14,22]. Most of the feeding dives (84%) in our study took place in the top 30 meters of the water column (19.2 m ± 20.3 SD), which is relatively shallow compared to other tagging studies [4,14,22]. Consistent with previous research, our recorded non-feeding dives were on average shallower (17.2 m ± 13.5 SD) [18]. Although the bathymetry is variable in Iqalujjuag Fjord (IQ), it is relatively shallow with a maximum depth of 137 meters. The physical characteristics of IQ may have limited the maximum foraging dive depth of whales in our study. In addition, IQ also has a complex biophysical environment, with freshwater inputs from rivers and glaciers, and complex depth stratified calanoid copepod prey layers that are heavily influenced by the tidal cycle [46]. Systematic active acoustic surveys (Simrad EK60 at 120 and 200 kHz transducers) carried out in conjunction with our tagging, showed that despite variability in the water column, three copepod prey layers were detected between 10-15m, 45-60m and 110-120m [46]. These prey layers correspond with the variability in the dive depth of tagged whales in our study with mean depths of confirmed foraging occurring at 19.16 meters (± 20.35 SD; Table 3 and S1 Fig).

### Dive shape

Dive shape is commonly used to discern putative feeding from non-feeding dives. U- and Square-shaped dives have relatively long bottom times and are associated with feeding behavior in multiple species [14,64–66]. During this prolonged bottom time, individuals ingest prey, and to make this energetically efficient, descent and ascent phases have a faster swimming speed compared to the bottom phase. V-shaped dives are likely exploratory in nature, where the animal travels to depth from the surface and back again [64]. In addition, V-shaped dives are generally shallower than U-shaped dives [4,18,64]. The majority (80%) of feeding dives (n = 50) in our study were Square-shaped, which is consistent with the assumption that Square- and U-shaped dives are indicative of feeding behavior. Similarly, 77.13% of all dives were found to be Square-shaped compared to 19.65% of U-shaped dives in KF during August 2016 [22]. However, in our study we found that 63% of confirmed non-feeding dives (n = 24) were also Square-shaped. Only 12.5% of non-feeding dives were classified as V-shaped, which are thought to be more exploratory dives and not associated with foraging activities. Consequently, the time spent foraging is likely overestimated if relying on shape alone. Dive phase classification is contingent upon accurately identifying the descent, bottom and ascent phases, which is inherently more challenging when dive depth and duration is variable. This is especially true for shallow dives, where bottom depth may reflect bathymetry (e.g., shallow coastline) or the depth of maximum zooplankton abundance.

Accurately identifying dive phases (ascent, descent and bottom) is challenging when depth and duration are variable. When manually selecting the start and end of the bottom phase of each dive in the Shiny app of the “bustR” package, only the TDR data was available. Several dives were relatively shallow and sometimes of short durations (e.g., < 3 minutes). Consequently, we encountered many instances where there was no clear start and end of the bottom phase, where the depth of the whale should plateau (Fig 3). This resulted in some dives missing a descent and ascent phase. Due to the difficulty of assigning the start and end of dive phases, particularly for shallow dives, it’s possible that the time spent during the bottom phase was overestimated resulting in an inflated number of U- and Square shaped dives assigned as non-feeding. If this is the case, it highlights an inherent challenge associated with using dive-shape to infer shallow feeding behavior.

This study underscores the value of integrating high-resolution biologgers with camera-validated observations to accurately quantify bowhead whale foraging behavior in dynamic environments with tidally driven shallow prey layers and deeper aggregations near the seafloor [46]. Using long-term satellite tags that transmit location and TDR data does not fully capture the feeding behavior of bowhead whales and may overestimate foraging efforts. Instead, time of day, average swim speed, body position and changes in mean swim velocity, captured foraging behavior of bowhead whales from a biomechanical point of view, which were validated by video footage. With climate change and the effect it has on the abundance and composition of *Calanus* spp. copepods, it is important to obtain robust estimates of foraging effort. Applying our kinematic predictors of feeding to unvalidated biologging data in the future, we will obtain estimates of foraging effort to predict daily energy intake, which can be balanced against energy expenditure to determine whether individuals are encountering an energetic imbalance due to climate mediated shifts in prey.

## Supporting information

S1 File Supporting information for Bowhead whale foraging dives are defined by speed and body orientation. (PDF)
