# Supplementary material for: Bowhead whale foraging dives are defined by speed and body orientation
Source: PLoS One. 2026 Apr 24;21(4):e0343408. doi: 10.1371/journal.pone.0343408 (PMC13108869; doi:10.1371/journal.pone.0343408)

Supplemental materials

Table S1. Overview of correlation coefficients of accelerometer jiggle with Orientation-Corrected Depth Rate (OCDR)

Deployment ID	***R-coefficient***
av230803-180a	*0.26*
av230803-P48_a	*0.50*
av230803-P48_b	*0.50*
av230805-P46_a	*0.34*
av230805-P46_b	*0.34*
av230806-P48	*0.95*
av230810-46	*0.63*
av240809-68	*0.74*
av240809-P47	*0.88*
av240812-48	*0.61*

Table S2. State and Point events recorded in Behavioral Observation Research Interactive Software (BORIS) from forward-facing video footage recorded by CATS tags. *State events represent longer periods of time behavior is displayed (e.g. "Foraging"). Point events are single moments in time behaviors are displayed.*

**Event**	**Description**	**Type of event**
**Start deployment**	Start of a new deployment	Point
**End deployment**	End of a deployment	Point
**Foraging**	Confirmed feeding, mouth is open	State
**Potential foraging**	Potential foraging, mouth is not clearly visible but based on behavioral clues feeding is suspected.	State
**Surface**	Whale is breaching the surface	State
**Rubbing**	Whale is rubbing rocks or against other conspecifics	Point
**Conspecifics**	New individual that is not tagged whale comes into frame	Point
**Copepods**	Copepods and other zooplankton species are visible on the CATS camera	State
**Coordinated foraging**	Multiple bowheads (including tagged individual) are foraging at the same time	State
**Fluke up**	The fluke is at its highest point	Point

Table S33. Summary of all kinematic variables calculated over the entire dive (whole dive) or dive phase (descent, bottom, ascent) calculated from CATS tags deployment.

Variable	Whole dive	Descent	Bottom	Ascent
Start and end time (UTC)	X			
Max depth (meters)	X			
Mean depth (meters)			x	
Depth variance (meters)			x	
Behavior observed in video audit	X	X	X	X
Duration (minutes)	X	X	X	X
Vertical displacement Rate (m/s)		X		X
Fluke stroke frequency (Hz)		X	X	X
Fluke rate (flukes/sec)		X	X	X
Jerk median (m/s^3)		X	X	X
Jerk mean (m/s^3)		X	X	X
Peak jerk (m/s^3)		X	X	X
Fluke rate change (fluke/sec)		X	X	X
VeDBA (m/s^2)		X	X	X
Roll (degrees)		X	X	X
Peak roll (degrees)		X	X	X
Pitch (degrees)		X	X	X
Heading variance (degrees)		X	X	X

Speed (m/s)		X	X	X
Change in speed (m/s)		X	X	X
Time of day (hours in UTC) sinus	X			

13

Table S4. Summary of all deployments where pitch, roll and heading could be measured. *Tag ID is made up of abbreviated species name (Arvik, Inuktitut name), date(YYMMDD)-tagnum_#num_deployment_date. Datetime and latitude and longitude (decimal degrees) are recorded from handheld GPS.*

**Tag ID**	**Date and Time (UTC)**	**Latitude (DD)**	**Longitude (DD)**	**Attachment type**	**Body length (meter)**	**Sex**	**Total deployment duration (hours)**	**Total video time (hours)**	**Used in GLMM analysis**
av230803-180a	2023-08-03 15:52	65.66787	-65.1102	Dermal		NA	0.36	0.10	Yes
av230803-P48_a	2023-08-03 15:21	65.66875	-65.1171	Suction		M	0.15	0.12	Yes
av230803-P48_b	2023-08-03 15:37	65.67024	-65.1078	Suction		NA	2.31	1.72	Yes
av230803-P49	2023-08-03 18:42	65.67066	-65.108	Suction	11.6	NA	4.27	NA	No
av230805-P46_a	2023-08-05 13:56	65.67248	-65.1139	Suction		NA	0.22	0.22	Yes
av230805-P46_b	2023-08-05 15:31	65.66419	-65.1184	Suction		M	3.74	2.66	Yes
av230805-P49_a	2023-08-05 14:34	65.66802	-65.1106	Suction		M	0.93	NA	No
av230805-P49_b	2023-08-05 18:22	65.67187	-65.1054	Suction	9.7	F	0.87	NA	No

av230806-P48	2023-08-06 16:07	65.6633	-65.1176	Dermal	10.8	M	24.35	1.32	Yes
av230806-P49a	2023-08-06 15:22	65.667	-65.1215	Suction		M	1.48	NA	No
av230806-P49b	2023-08-06 18:04	65.6677	-65.1116	Dermal		NA	1.36	NA	No
av230810-46	2023-08-10 13:53	65.6712	-65.1102	Suction	9.4	NA	2.50	0.60	Yes
av240809-68	2024-08-09 19:02	65.701918	-65.048831	Suction		M	0.61	0.61	Yes
av240809-P47	2024-08-09 18:05	65.671	-65.116922	Dermal		M	14.05	4.87	Yes
av240812-48	2024-08-12 18:47	65.67153	-65.11588	Suction		NA	0.35	0.35	Yes

Fig. S1. Time-Depth recorder from CATS tags. *Green shading reflects period when the camera was recording. Pink values represent time points when feeding confirmed to occur based on video audits (mouth open). Purple points show instances of confirmed non-feeding was audited in BORIS.*

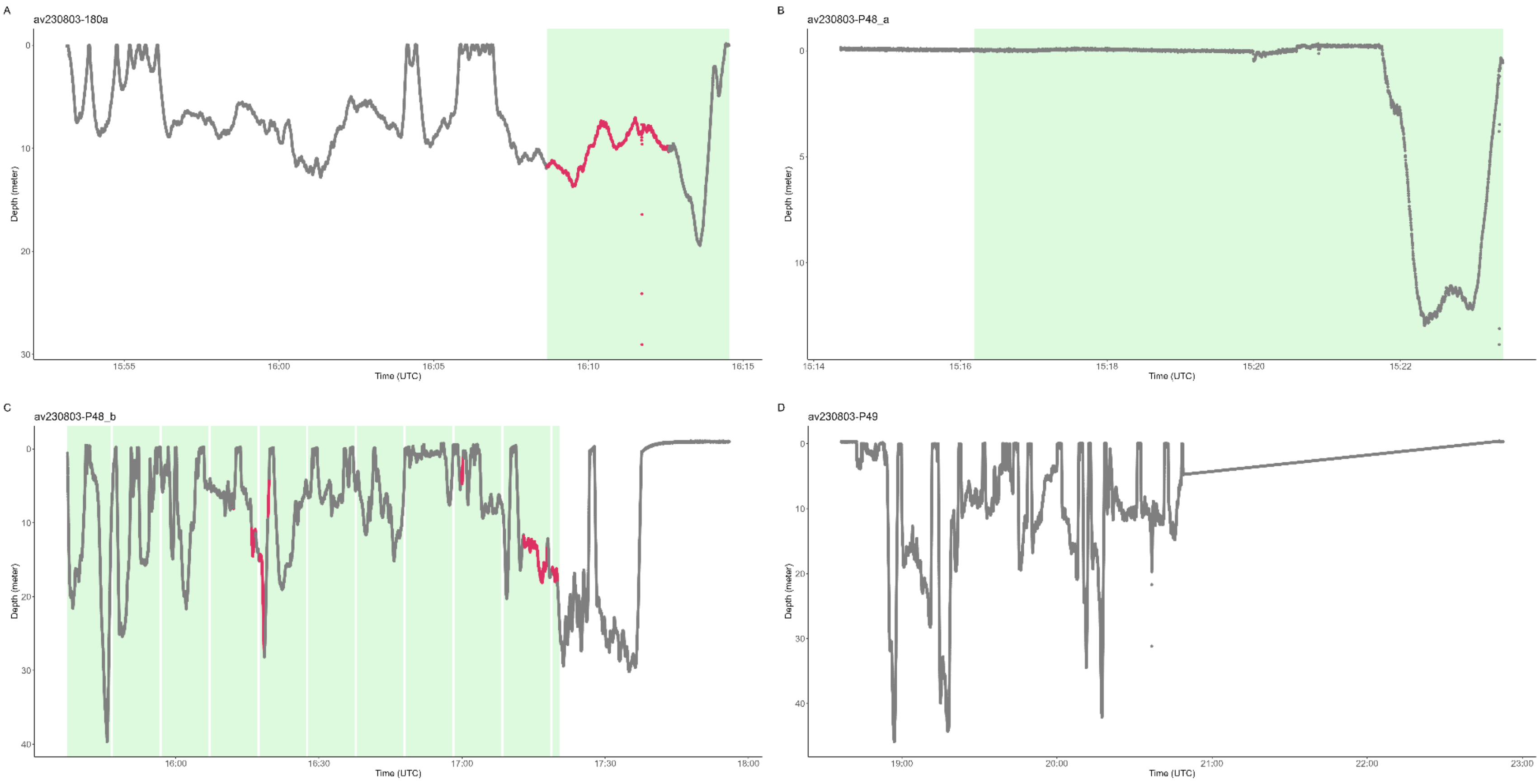

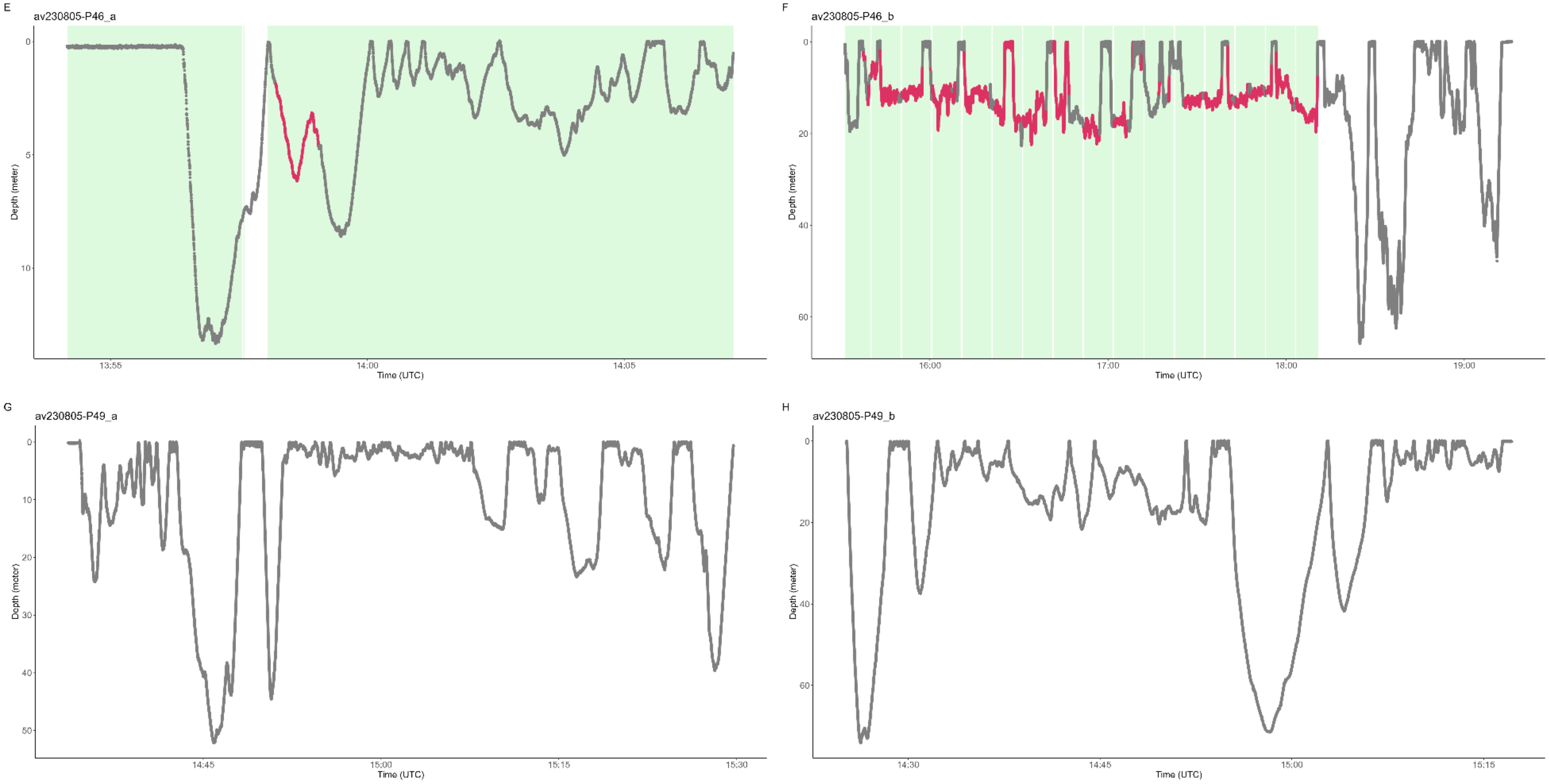
E
av230805-P46_a
Depth (meter)
Time (UTC)
13:55
14:00
14:05
F
av230805-P46_b
Depth (meter)
Time (UTC)
16:00
17:00
18:00
19:00
G
av230805-P49_a
Depth (meter)
Time (UTC)
14:45
15:00
15:15
15:30
H
av230805-P49_b
Depth (meter)
Time (UTC)
14:30
14:45
15:00
15:15

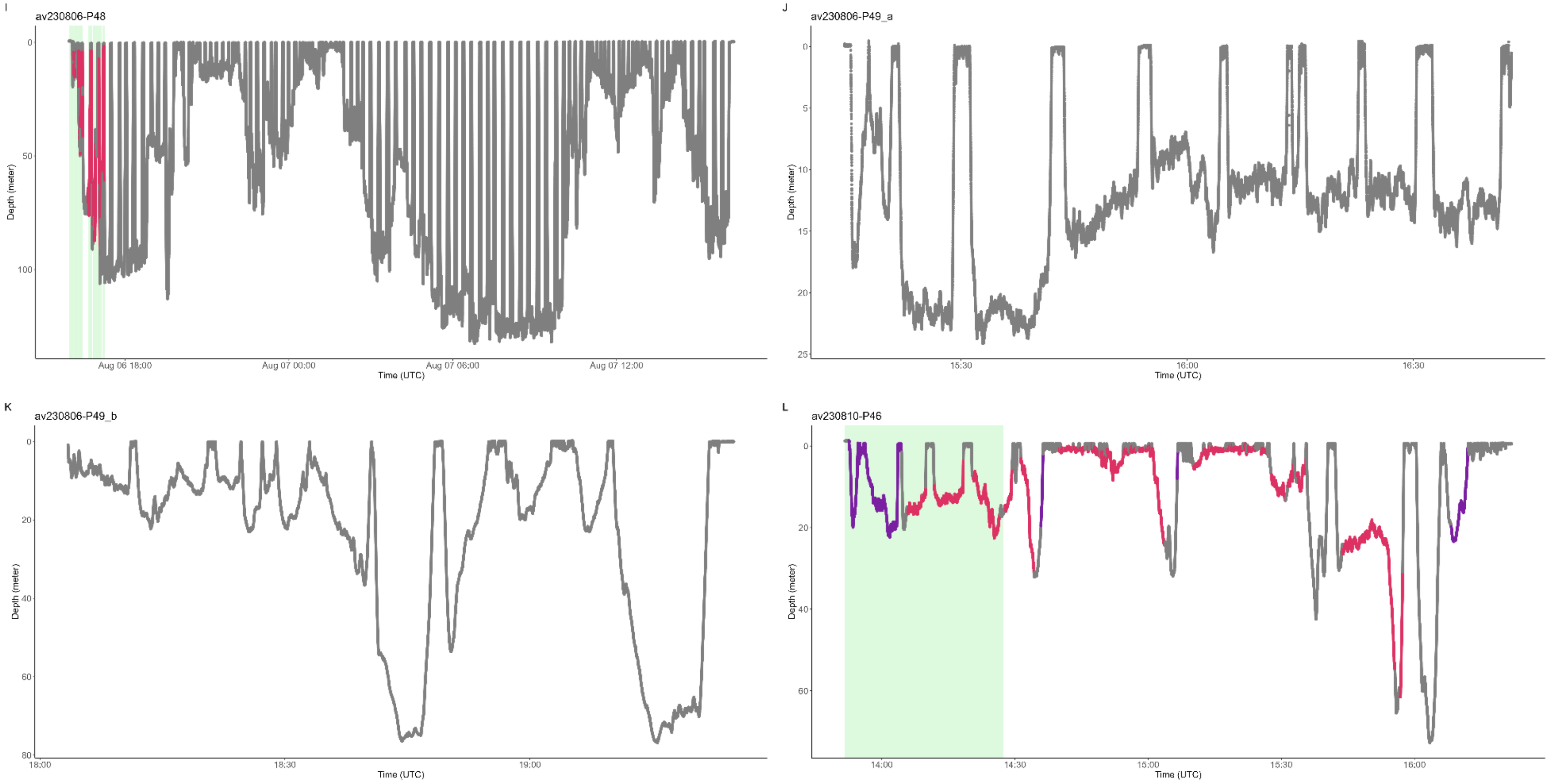
I
av230806-P48
Depth (meter)
Time (UTC)
Aug 06 18:00
Aug 07 00:00
Aug 07 06:00
Aug 07 12:00
J
av230806-P49_a
Depth (meter)
Time (UTC)
15:30
16:00
16:30
K
av230806-P49_b
Depth (meter)
Time (UTC)
18:00
18:30
19:00
L
av230810-P46
Depth (meter)
Time (UTC)
14:00
14:30
15:00
15:30
16:00

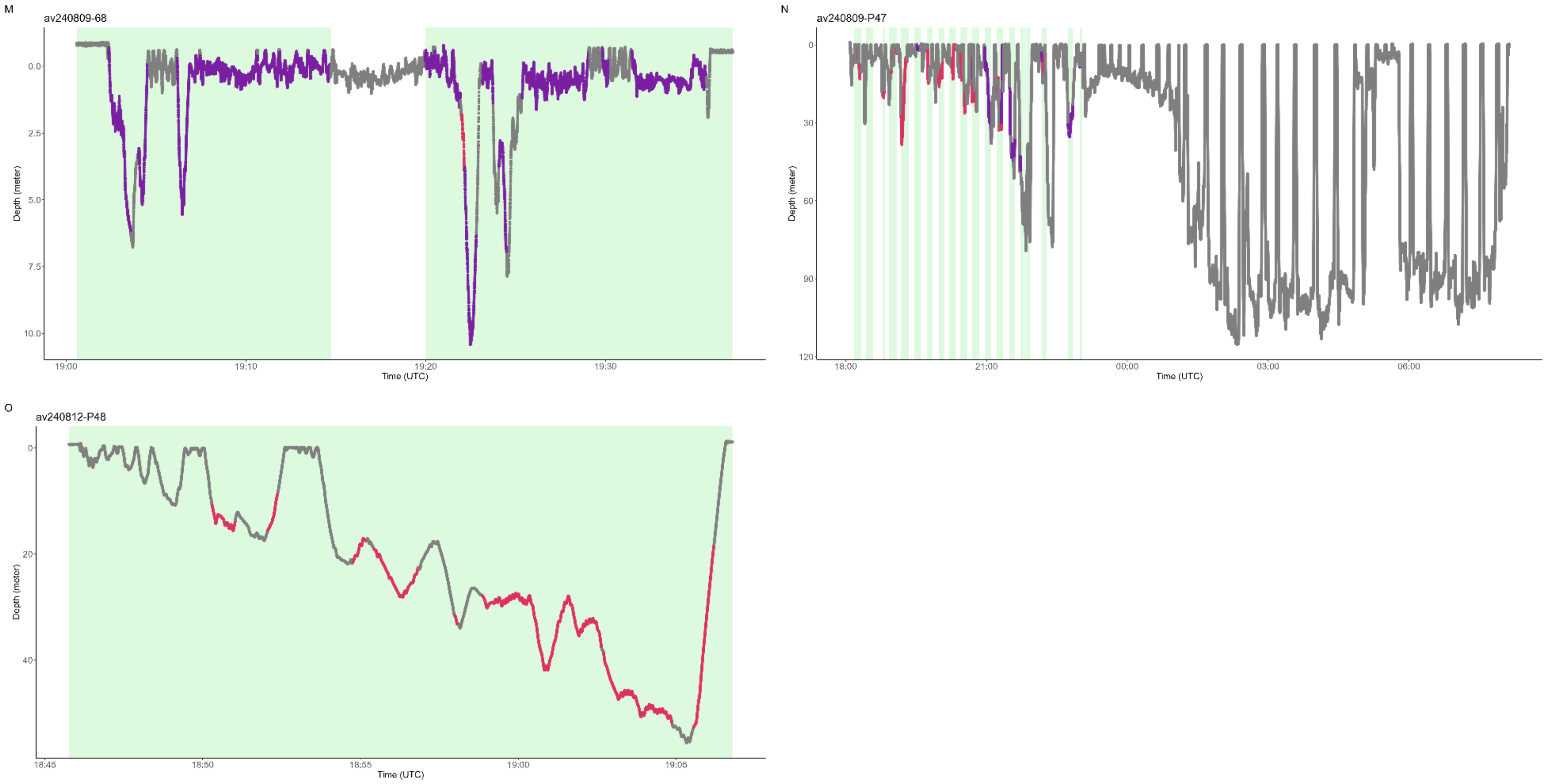
M
av240809-68
Depth (meter)
0.0
2.5
5.0
7.5
10.0
19:00
19:10
19:20
19:30
Time (UTC)
N
av240809-P47
Depth (meter)
0
30
60
90
120
18:00
21:00
00:00
03:00
06:00
Time (UTC)
O
av240812-P48
Depth (meter)
0
20
40
18:45
18:50
18:55
19:00
19:05
Time (UTC)

Fig. S2. Example of a deep dive of tagged whale. Example of confirmed non-foraging dive on August 7th, 2023. *Top panel is an overview of the Time-Depth Recorder, which is colored for the speed of the animal, where green is lower and red are higher values for speed (m/s). The middle panel is an overview of the body orientation of the individual, where (body) Roll, Pitch and Heading are given in degrees. The lower panel give an overview of movement variables: jerk (m/s^3) and fluke stroke (degrees per second, based on y-axis gyroscope). Tag ID: av20230806-P48*

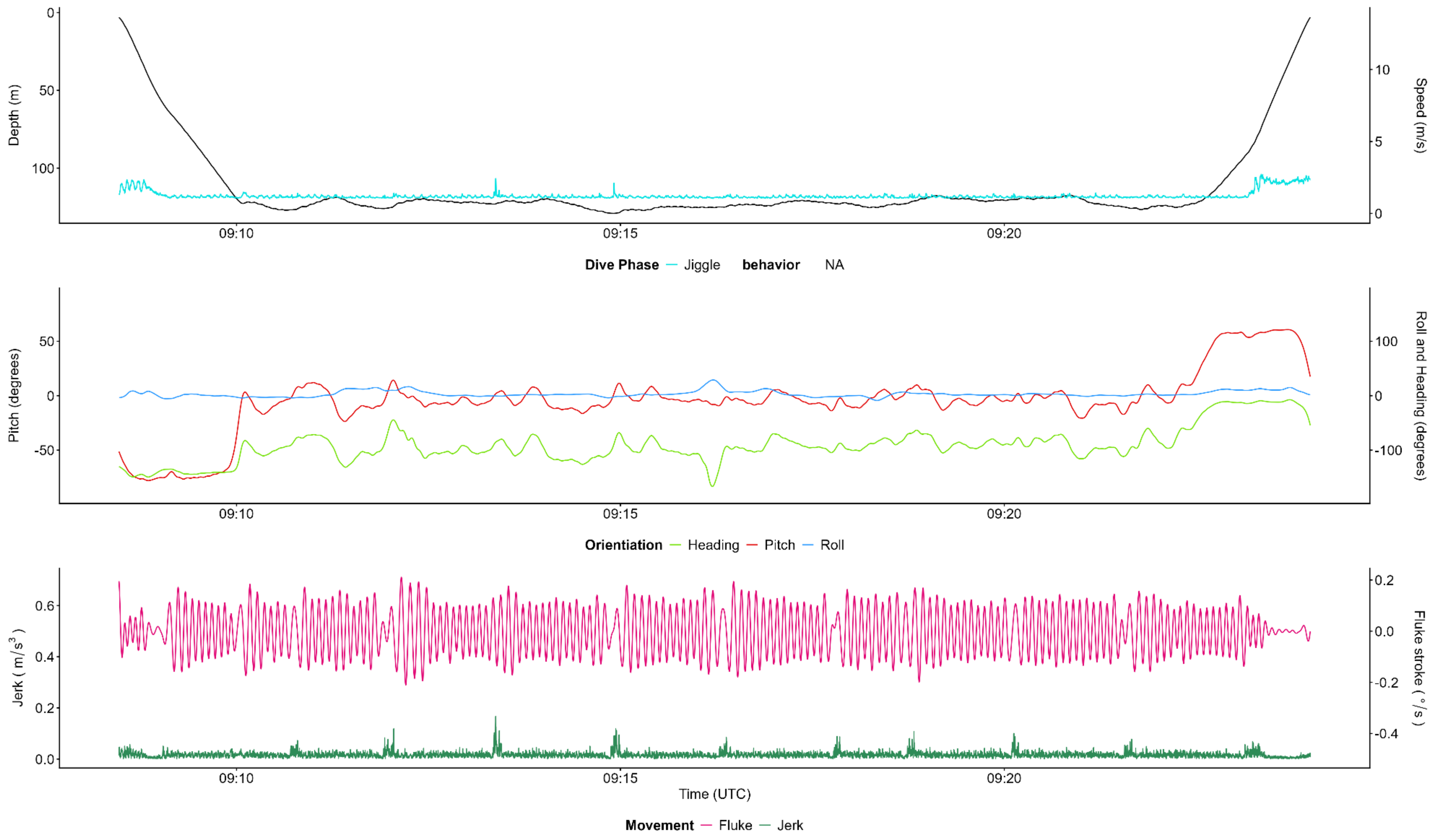

Supplement: S1 File — (PDF) [file pone.0343408.s001.pdf]
